# Understanding the factors regulating host–microbiome interactions using *Caenorhabditis elegans*

**DOI:** 10.1098/rstb.2023.0059

**Published:** 2024-05-06

**Authors:** Anupama Singh, Robert J. Luallen

**Affiliations:** Department of Biology, San Diego State University, San Diego, CA 92182, USA

**Keywords:** *Caenorhabditis elegans*, microbiome, factors, genetics, commensals

## Abstract

The Human Microbiome Project was a research programme that successfully identified associations between microbial species and healthy or diseased individuals. However, a major challenge identified was the absence of model systems for studying host–microbiome interactions, which would increase our capacity to uncover molecular interactions, understand organ-specificity and discover new microbiome-altering health interventions. *Caenorhabditis elegans* has been a pioneering model organism for over 70 years but was largely studied in the absence of a microbiome. Recently, ecological sampling of wild nematodes has uncovered a large amount of natural genetic diversity as well as a slew of associated microbiota. The field has now explored the interactions of *C. elegans* with its associated gut microbiome, a defined and non-random microbial community, highlighting its suitability for dissecting host–microbiome interactions. This core microbiome is being used to study the impact of host genetics, age and stressors on microbiome composition. Furthermore, single microbiome species are being used to dissect molecular interactions between microbes and the animal gut. Being amenable to health altering genetic and non-genetic interventions, *C. elegans* has emerged as a promising system to generate and test new hypotheses regarding host–microbiome interactions, with the potential to uncover novel paradigms relevant to other systems.

This article is part of the theme issue ‘Sculpting the microbiome: how host factors determine and respond to microbial colonization’.

## 1. Introduction

Microbes are evolutionarily the oldest and the most abundant life form, believed to colonize all metazoans and plants through symbiotic, pathogenic or commensal-like associations. The term microbiome is used for all the resident microbes, including bacteria, fungi and viruses, in any given biotic or abiotic habitat. Microbiomes are known to play fundamental roles in many biological processes, from being required for development, immunity, metabolism, organ function and behaviour, to causing disease and death.

Until very recently, the microbiota was commonly referred to as ‘the forgotten organ’ [1]. However, even though the study of animal microbiomes is perceived as a young discipline, we are already using probiotics and faecal microbiota transplant procedures to manage health complications and diseases in humans [2–4]. The Human Microbiome Project (HMP) conducted between 2007–2016, took a comprehensive, multidisciplinary approach to characterize the human microbiome from different organs of healthy and diseased North Americans [5,6]. The studies from the HMP characterized the composition and abundance of microbes from different organs of healthy individuals [7,8]. Additionally, these studies discovered that microbial compositional changes in different organs are associated with specific diseases, including gut microbiome changes associated with inflammatory bowel disease, nasal and gut changes associated with pre-diabetes, and vaginal microbiome changes associated with preterm births [9]. In addition to creating a wealth of knowledge and community resources, this decade long research initiative also identified potential gaps and limitations [6].

The HMP opened up a multitude of research avenues to understand the interactions between an animal host and its microbiota, including the development of new tools and techniques in genetically amenable model systems such as *Caenorhabditis elegans* and *Drosophila melanogaster.* A causal role of human genetics in shaping the gut microbiome is generally difficult to investigate [10,11], and was an understudied aspect of the HMP, as the human subjects belonged to different ethnicities with limited genetic diversity. Over the last 70 years, *C. elegans* has been extensively used to decode genes and pathways that regulate complex organismal phenotypes, including development, neurobiology, health, ageing and pathogenesis. *Caenorhabditis elegans* has several traits that makes it a suitable model to study host–microbiome interactions, including a short lifespan, genetic tractability and well-defined tissue systems [12]. Additionally, *C. elegans* is transparent throughout its life, allowing for easy visualization of microbial colonization using simple microscopes, with sufficient spatial resolution [13,14]. They can also quickly and easily be made germ-free by using a rather simple bleaching method. These properties make *C. elegans* suitable for studying its interaction with a single microbial species or more diverse microbial communities [15,16]. However, until recently, the vast majority of studies in *C. elegans* were conducted in the absence of a microbiome. Generally, *C. elegans* is conventionally grown on the *Escherichia coli* strain OP50 as a sole food source, which does not colonize the gut of healthy, young animals [17].

The recent extensive worldwide sampling of wild *Caenorhabditis* nematode populations has led to the isolation and identification of a vast array of microbes naturally associated with these animals [18]. In their habitat of rotten fruits and plant matter, *C. elegans* and related nematodes are now known to interact with a variety of obligate and non-obligate pathogens, such as bacteria, viruses, fungi and parasites [18–24]. More recently in 2016, three parallel studies provided the first description of the *C. elegans* microbiome and found that it assembles a gut microbiome in a deterministic, non-random manner, which is distinct from the microbiome of its habitat ([25–27], and reviewed in [28]). These discoveries were combined to define a core intestinal microbiome of *C. elegans*. Initially a set of 12 bacteria from nine different families (called CeMbio) were chosen based on taxonomic diversity, capacity for easy *in vitro* culture and high prevalence in microbes associated with wild-isolated *C. elegans* [15]*.* Later a set of 51 bacteria from 10 different families were added to the core CeMbio (called BIGbiome) for more comprehensive studies [16]. In addition to CeMbio and BIGbiome, several individual bacterial species found in wild nematodes also allow for direct mechanistic studies of host–bacterial interactions [13,16,29,30]. For example, our laboratory has isolated several new species of adhering microbiome bacteria with the goal of dissecting host and bacterial pathways that regulate niche formation in the gut [14,31]. The genetic amenability of *C. elegans* and their bacterial microbiome, coupled with the ease of implementing complex experimental designs and large-scale genetic screens, makes it an excellent model for dissecting the genetic basis of host–microbiome interactions. In addition, *C. elegans* is ideal for studying the impact of the microbiome on host fitness, tissue-specific health outcomes, metabolism and behaviours, using large genetically homogeneous populations.

This review provides an analysis of different factors that regulate the host–microbiome interactions, including behaviour, immunity, diet, and the gut niche, with a focus on studies conducted in the *C. elegans* model. In addition, the review underlines the role of host and microbial genes in shaping the microbiome, as well as the amenability of the *C. elegans* holobiome to unravel yet unknown genetic determinants that drive host–microbiome associations. In the end, this review identifies some of the understudied aspects of host–microbiome research that can be tackled in *C. elegans*.

## 2. Understanding factors regulating host–microbiome interactions

Host–microbe interactions are expected to be shaped by a complex interplay between several abiotic and biotic factors. The impact of a microbe on host fitness and health is dynamic and context-dependent, and defines whether the relationship is pathogenic, mutualistic or commensal-like. Besides this, the composition and abundance of a microbial community is shaped by inter-species microbial competition and cooperation. In this review, we explore factors that have been shown to regulate host–microbiome interactions in *C. elegans*. In §2a, we discuss how microbial odours and metabolites can influence host behaviours for selection of certain host–microbe associations. In §2b, we describe how microbiota are critical for optimal development of host immunity and pathogen resistance. In §2c, we analyse several microbiota-derived vitamins and metabolites, otherwise unobtainable for the host, that are essential for host function and fitness. Finally, in §2d, we underline niche-specific selection pressures, especially in the gut, that are exerted by abiotic factors such as pH and oxygen conditions, and adhesion factors and receptors that confer specificity and stability to various host–microbiota interactions.

### (a) Microbe-seeking and microbe-driven behaviours: stay, avert or hitchhike

How microbial associations and microbe-derived metabolites modify animal behaviours is an emerging area of study in host–microbiome interactions [32]. Can the gut microbiota influence what we eat and manipulate how we behave? The answer is, yes! Gut microbiota-derived metabolites and neurohormones can manipulate eating behaviours by impacting satiety [33], and can even modify the sense of taste and food cravings through different mechanisms [32]. In addition, human gut microbiota-derived metabolites such as short-chain fatty-acids (SCFAs) can directly induce the secretion of gut-derived hormones that indirectly modulate brain functions via the vagal nerve in the gut-brain axis, resulting in an impact on behaviour [32,34]. Interestingly, a recent study demonstrated the therapeutic potential of butyrate (an abundant SCFA) and human butyrogenic bacteria in ameliorating the toxic aggregation of metastable proteins induced by human enteric pathogens, using the *C. elegans* model [35].

**Figure 1.**
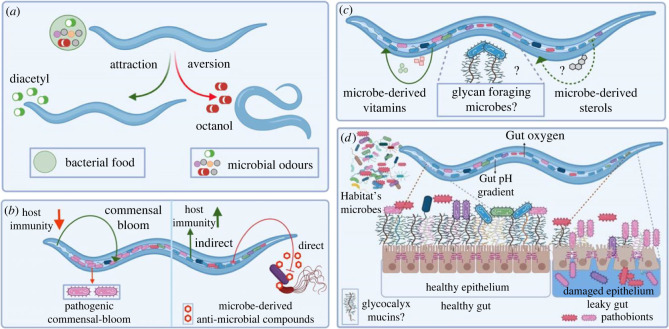
Different host and microbial factors shape the microbiome and regulate its homeostasis. (*a*) Microbial odour driven attraction and repulsion regulates food-seeking behaviour in the host. (*b*) Host and microbe-derived immunity together sculpt the microbiome. (*c*) Unique metabolic traits of the host and their microbes provides for an optimal nutrition. (*d*) Intestinal factors select and regulate the gut microbiome homeostasis.

In the wild, *C. elegans* is exposed to a variety of microbes, from pathogens to commensals. Given that *C. elegans* are bacterivores, many microbes also serve as food sources. It is likely that *C. elegans* uses an innate sense of smell to quickly profile its habitat and make immediate decisions, including foraging, avoidance, eating or egg-laying [36]. In addition to these innate behaviours, worms are also capable of long-term learned behaviours elicited by changes in their internal state [37,38]. As a result, while ingestion of nutritive bacteria leads to learned attraction for exploitation of a bacterial food source, ingestion of pathogens and toxins leads to learned aversion for avoiding stress and damage ([39–41]; figure 1*a*).

Microbial-derived odours are expected to impact seeking and ingestion of a food source by *C. elegans*. Interestingly, several bacteria from the natural habitat of *C. elegans* were found to release odours that include previously known attractants and repellents, studied for decades in *C. elegans* neurobiology and behaviour [42–44]. For example, *Lactobacillus paracasei* were isolated from rotten citrus fruits that also harboured wild *C. elegans.* This species of bacteria was found to naturally produce the chemical diacetyl, a known attractant of *C. elegans* [45]. This attractant is a volatile byproduct of citric acid metabolism by *L. paracasei*, and while this bacterium does not appear to colonize *C. elegans,* it possibly attracts the nematodes to rotting citrus fruits that are rich in other microbes [16].

In yet another interesting mechanism, a known *C. elegans* commensal bacterium*, Providencia alcalifaciens* (strain JUb39) [16], uses sensory attraction and sensory override mechanisms to manipulate *C. elegans* feeding behaviours. *Providencia alcalifaciens* releases isoamyl alcohol, another well-studied *C. elegans* attractant [44], that possibly makes this bacterial species a preferred food choice over the standard *E. coli* diet [46]. Then, on colonizing the *C. elegans* gut, *Pr. alcalifaciens* uses host-derived tyrosine to produce the neurotransmitter tyramine that is then used by *C. elegans* to synthesize octopamine, thus subverting the need for the host to produce tyramine. Thus, bacterial tyramine uses *C. elegans* octopamine signalling to essentially manipulate *C. elegans* into selecting a food source that is beneficial for both of them [46,47].

In an example of beneficial selection by the host, *C. elegans* were colonized on being attracted to a beneficial *Pantoea* strain of bacteria, that hindered the pathogenic colonization by *Pseudomonas aeruginosa* [29]. These observations imply a possible habitat-driven coevolution between this beneficial *Pantoea* sp. and *C. elegans,* as these are bacteria found in the same natural habitat with nematodes, and host colonization by beneficial commensals probably block infection by pathogens.

How does the microbiome shape *C. elegans* behaviour during food scarcity? Food deprivation is known to elicit behavioural changes from enhanced risk-taking during food search [48], to alternate developmental decisions, such as dauer formation. During unfavourable conditions including food scarcity, instead of further development into reproductive adults, *C. elegans* larva develop into a metabolically quiescent state called the dauer that is capable of surviving for prolonged periods [49]. Consistently, dauers are very prevalent in the natural habitat of *C. elegans*, and are probably the first to enter and the last to exit a new food source in a given habitat [18,21]. Dauers exhibit a phoretic behaviour called nictation, that enables their dispersal to geographically isolated habitats by hitchhiking on snails and isopods [18,21]. Interestingly, trimethylamine, a human gut flora metabolite was shown to mediate food-sensing behaviour in *C. elegans* and regulate dauer formation [50]. However, it remains unknown if microbial odours or metabolites derived from the *C. elegans* microbiota impact their dauer entry and exit decisions, or if dauer-associated microbes define the microbial communities in a new habitat. This is a fascinating area for future research in *C. elegans*, as it is likely that many host–microbiome co-evolutionary processes have occurred with the dauer state. Given that the dauer stage probably acts as a transport mechanism for some bacteria, dauers could possibly help in the survival of resident microbes between discontinuous food sources [51] and across geological time scales through suspended metabolism (cryptobiosis) [52].

### (b) Microbe-derived immunity together with host's immunity sculpt the microbiome: from commensal-like to a pathobiont-like relationship

The microbiome is known to play a major role in the development and maintenance of host immunity. In parallel, host immunity has been identified as a common and critical factor in shaping the microbiome [53]. Especially, the composition of the first human microbiota is critical for the development and priming of the neonatal immune system. Vaginal births are known to result in better vertical transmission of microbiomes and greater gut microbiota diversity compared to cesarean delivery [54], which subsequently impact childhood pathologies and immune-mediated diseases [55]. Likewise, in the absence of gut microbiota, germ-free mice have morphological and functional defects in the development of adaptive immunity and regulation of immune cells [56].

The natural *C. elegans* microbiota are also known to contribute to host immunity, either directly or indirectly to counteract pathogens ([27,57]; figure 1*b*). Similarly, host immunity also impacts the microbial proliferation and composition [13]. Together, this can be conceptualized as the collective immunity of the host and its microbiome, which for *C. elegans* includes multiple innate immunity pathways combined with acquired immunity from the associated microbes that together dictate the carrying capacity and composition of its microbiome.

The microbiome can contribute to host immunity by directly counteracting pathogens. This occurs via inter-species or inter-kingdom interactions between microbes that shape the microbiome through cooperation or competition, sometimes through microbial derived products with anti-microbial effects [26]. For example, *Pseudomonas lurida*, strain MYb11, provides broad-range protection to *C. elegans* against the fungal pathogen *Drechmeria coniospora* [26], Gram-negative *Ps. aeruginosa*, and Gram-positive *Bacillus thuringiensis* bacteria [58]. *Pseudomonas lurida* was found to directly antagonize *Bacillus thuringiensis* growth by generating massetolide E, a cyclic lipopeptide surfactant of the viscosin group, which has a bacteriostatic effect on the pathogen*.* As such, worms colonized by *Ps. lurida* exhibited significant improvement in epithelial barrier function (leaky gut) induced by *Bacillus thuringiensis* ([58]; figure 1*b*). *Pseudomonas lurida* benefits its host in multiple ways, however gut colonization results in a small reduction in *C. elegans* lifespan, suggesting that there are some trade-offs to the host when colonized by these protective bacteria [59].

The microbiome can also contribute to host immunity by indirectly counteracting pathogens, through modulation of host immunity and stress pathways that regulate infection [27,30,60]. *Pseudomonas fluorescens*, strain MYb115, protects *C. elegans* without reducing the pathogen load of *Bacillus thuringiensis*. Therefore, it is hypothesized that *Ps. fluorescens* helps *C. elegans* cope with infection, possibly by enhancing damage repair in the gut epithelium [58]. This idea is supported by the fact that *Ps. fluorescens* decreases the epithelial barrier dysfunction induced by *Bacillus thuringiensis* infection [58], potentially by altering host lipid metabolism, and cytoskeleton reorganization through intermediate filaments [61], which are known to protect against microbial insults [62]. The impact of *C. elegans* microbiome on cytoskeleton reorganization and epithelial-barrier function requires further investigation.

The capacity of some microbiome bacteria to activate host immunity pathways, however, may be a result of their cryptic virulence, as they are capable of becoming pathogenic when host immunity is not activated. This was recently found to be the case for a large majority of the *C. elegans* CeMbio bacteria that induce the helix-loop-helix-30/transcription factor EB immune pathway, which were pathogenic when this pathway was knocked out, suggesting that these bacteria have characteristics of pathobionts (commensals with pathogenic potential) [63]. Consistent with this idea, the state of host immunity can also impact the composition of the microbiota. For example, the known *C. elegans* commensal bacteria, *Enterobacter cloacae,* strain CEent1, is known to protect from gram-positive *Enterococcus faecalis* infection [57]. However, *Enterobacter cloacae* itself becomes a pathogen in immune-compromised *C. elegans* strains with loss-of-function mutations in the transforming growth factor β/bone morphogenetic protein pathway, a conserved development and immunity pathway [13,64,65]. This rise in pathogenicity is evidenced by a selective *Enterobacter* spp. bloom that shortens host lifespan with enhanced susceptibility towards *Enterococcus faecalis* (figure 1*b*).

It is therefore evident from these studies in *C. elegans*, that both host immunity and microbe-derived immunity dictate microbiome composition, and a decline in host immunity plays a role in the shifting commensal-like microbes to pathogens (figure 1*b*). However, it is also possible that some microbes can actively switch from commensal-like to pathogenic traits through changes in their gene expression to produce toxins or other harmful metabolites [66], an aspect of the *C. elegans* microbiome that needs investigation.

### (c) Diet and metabolism: a nourishing factor in host–microbiome interactions

Host–microbiota assemblies are expected to be governed by the availability and distribution of nutrients in a given habitat. For microbiome bacteria, it is expected that the bulk of nutrition comes from the host. However, microbes reciprocally allow their host to use resources that they otherwise cannot digest or metabolize on their own, including essential vitamins. Consistent with this idea, the lack of a metabolically active gut microbiome causes caloric restriction in germ-free mice [67], and axenically grown *C. elegans* [68], showing a host's dependence on their microbiome for optimal nutrition. Similarly, our diet can dramatically impact our microbiome, as a Mediterranean-style diet rich in fruits and fibres, and low in fat, can have a positive impact on the gut microbial diversity and metabolism [69–71]. By contrast, a Western-style diet low in fibre and high in fat, can result in reduced levels of beneficial bacteria with low diversity [72], resulting in obesity and altered behaviours [73].

*Caenorhabditis elegans* are bacterivores and thus have a more complicated relationship with their microbiota, which can also serve as a nutrition source. As such, it is not so straightforward to separate the non-dietary commensal- or pathogen-like impact of microbes from their potential dietary (or nutritive) roles. We have only begun to understand the impact of microbiome derived metabolites on *C. elegans* life-history traits. For example, of the several known essential nutrients commonly required by both humans and *C. elegans* [74], vitamin B12 is an example of a microbiota-derived essential cofactor whose deficiency leads to slow growth (figure 1*c*). Among the known members of the *C. elegans* microbiome, several *Pseudomonas* and *Ochrobactrum* species have genes in the biosynthetic pathway of vitamin B12 [75]. Without B12, *C. elegans* exhibit stunted development, loss of fertility, and reduced lifespan [76], as the cofactor is required in the methionine/S-adenosyl methionine cycle for converting homocysteine to methionine [77]. As another example, *C. elegans* are cholesterol auxotrophs as they lack the first three enzymes in the canonical cholesterol biosynthesis pathway. However, it was recently found that these animals are capable of converting dietary sterols obtained from fungi and plants into cholesterol [78], making it possible that microbiome bacteria or fungi can provide sterols for cholesterol production in *C. elegans,* an area that remains to be investigated (figure 1*c*). Besides providing for nutrients and essential vitamins, microbiota can also impact host metabolism. For an example, a known *C. elegans* commensal, *Ps. lurida* (MYb11) was found to increase vitellogenin protein (yolk protein) production in young *C. elegans* adults, believed to hasten their reproductive timing [61].

In addition, it is predicted that *C. elegans* microbiome bacteria use their other unique metabolic competencies to colonize and flourish in the gut. Along these lines, a recent study identified two key metabolic traits in known *C. elegans* commensals, namely, the ability to ferment pyruvate to acetoin which probably influences gut colonization, and the ability to degrade hydroxyproline which probably correlates with *C. elegans* population growth [75]. The fermentation of pyruvate to acetoin releases diacetyl, whose buttery odour is known to attract *C. elegans* and promote feeding behaviour [79], which would result in enhanced bacterial uptake and colonization. Similarly, hydroxyproline degradation is known to produce reactive oxygen species, which could induce *C. elegans* growth and reproduction, or alternatively bacteria degrading hydroxyproline can proliferate by using the amino acids as a carbon source [75].

Finally, an interesting aspect of host–microbiome associations is the ability of hosts to provide niche-specific food to microbiota species that are uniquely adapted to forage on them. In mammals, the mucus layer, in addition to its role as a protective physical barrier for the epithelia, is also known to provide nutrition to its microbes. Mucus is made of heavily glycosylated mucin proteins which can become a foraging avenue for glycolytic microbes that metabolize the complex glycosylated moieties to release sugars for their consumption [80]. The production, diversification and maintenance of a mucus layer is akin to a metabolic collaboration between the host and their microbiota. Consistent with this idea, germ-free mice have an underdeveloped mucus layer [81], and glycan metabolism is known to shape the human gut microbiota [80]. Several prominent human gut commensals include mucin-degrading bacteria, such as *Akkermansia muciniphila,* that can utilize mucin as their sole source of carbon and nitrogen (reviewed in [82]). Similarly, *Bacteroides thetaiotaomicron* exhibit adaptive mucus foraging to utilize glycan sugars in the absence of dietary polysaccharides [81], thereby contributing to the metabolic homeostasis in the gut. However, in *C. elegans*, we do not yet know whether its microbiota can induce glycan and mucin production, nor whether there are mucolytic and glycolytic foragers in its microbiota (figure 1*c*). Overall, for successful colonization, microbes likely integrate their metabolism with host metabolism to provide an optimal growth condition for both.

### (d) Mucins, epithelial barrier and intestinal factors: shaping the confines of the gut niche

Anatomical and tissue-specific factors are expected to drive the community composition of a microbiome, and here we focus specifically on the animal gut. In mammals, the gut is compartmentalized along the length, into the stomach, small intestine (duodenum and ileum), and the large intestine, each having unique structure and function. The different microbial composition in these tissues is predominantly dictated by their niche-specific environments, such as pH, oxygen, nitrate and bile acid metabolites (reviewed in [83]). Microbial interactions in the gut mainly occur with the secreted mucus layer or the glycocalyx, a layer of heavily glycosylated proteins (including membrane-bound mucins) at the apical surface of the epithelia. These coats create a protective barrier between the underlying gut epithelium and microbes. Besides being the first-line of defence, the mucus layer can provide substrate for bacterial adhesion, with host mucins bound by bacterial fimbriae or pili [84]. Research suggests that there is a potential cross-communication between the host gut and the microbiota. In fact, altered mucous glycosylation can impact the microbiota composition and intestinal architecture [85]. In turn, microbes are known to induce host secretion of mucus. This idea is supported by the ability of the mucolytic human gut commensal, *Bacteroides thetaiotaomicron*, to induce mucin secretion by regulating the differentiation of goblet cells [86].

*Caenorhabditis elegans* has a simpler and less compartmentalized alimentary canal. It begins with the pharynx, containing a neuro-muscular grinder that is very efficient in masticating bacteria. The grinder empties into the worm gut, an epithelial tube made of 20 polarized epithelial cells [87,88]. In order to colonize *C. elegans,* microbes must first survive mastication in the grinder. Then, they must adapt to the lumenal pH and oxygen conditions in the gut, while avoiding getting flushed out by gastric motility and defecation. In the gut, some bacteria can directly adhere to the intestinal epithelial cells to create a niche, whereas others remain sessile by persisting and/or replicating in the intestinal lumen without direct adherence ([31]; figure 1*d*). The *C. elegans* intestinal lumen is suggested to be aerobic, as their gut microbiota includes several known obligate aerobes, but is also colonized by several fermenting bacteria [75]. Additionally, the pH of the intestinal lumen of *C. elegans* is weakly acidic and discontinuous [89]. The low intestinal pH of the gut is a possible selection force, also known to modulate inter-microbial interactions during community assembly [90]. In turn, microbes can also condition the *C. elegans* intestinal environment to maintain pH gradients that are critical for the function of pH-dependent intestinal transporters [68]. In fact, the intestinal pathogens *Ps. aeruginosa* and *Enterococcus faecalis* were shown to neutralize the intestinal pH of the lumen upon infection [91], suggesting that microbiome bacteria are likely to play a role in pH alterations upon colonization.

Bacterial adhesion to host mucus glycans is a potential host selection mechanism that permits their gut retention and colonization [92]. Currently, we still do not know if *C. elegans* contains a flushable mucus layer, similar to other mammals, but we have visualized a potential mucus-like layer on intestinal epithelial cells that may be thick glycocalyx containing mucin-like proteins [93]. While many gut commensals in *C. elegans* have not been shown to colonize by adherence [21], a few are emerging with obvious direct interactions with gut epithelial cells [14,31]. A possible role of *C. elegans* mucins in microbial adherence remains understudied and is an active area of investigation. It is possible that in *C. elegans*, the heavily glycosylated glycocalyx can contribute to the adherence of gut commensals, and the gut microbiota in turn induce mucin-like protein secretion in the glycocalyx, somewhat similar to exchanges seen in mammals. In fact, a recent study has shown a requirement for the *C. elegans* mucin-like gene, *mul-1*, in *Ps. aeruginosa* pathology [94], and *mul-1* was previously shown to be upregulated during *Ps. aeruginosa* infection [95,96]. Super-resolution electron microscopy can be used to visualize the impact of *C. elegans* gut microbiota on the mucin-glycocalyx layer, and the integrity of underlying microvilli and brush border assembly [97,98]. In addition, *in vivo* molecular imaging of *C. elegans* mucin-type *O*-glycans can be implemented to understand how microbes drive the mucobiology of the *C. elegans* gut [99].

There are other known factors that impact the homeostasis of the gut microbiome, its mucus layer and the underlying epithelium. Age-related decline in the grinder efficiency and defecation frequency results in more live bacteria entering and proliferating in the gut, leading to constipation and death [17]. In fact, the standard *C. elegans* diet bacteria, OP50, are efficiently destroyed in the grinder of healthy, young animals, but can colonize the intestinal lumen in aged animals [100]. In addition, age-related depletion of the mucus-like layer and decline in gut barrier function is known to enhance intestinal leakiness and gut dysbiosis, which negatively impacts overall health and lifespan ([101,102]; figure 1*d*). Different members of the microbiota are known to impact the gut epithelium, with some pathogens disrupting it and a number of commensals fortifying it [103]. For example, several known commensal bacteria in *C. elegans* have been shown to improve the gut barrier function following pathogenic infections that would increase gut leakiness [58,61]. However, how *C. elegans* commensals modify gut mucin layer and the underlying epithelium is not well explored. In *C. elegans*, the impact of microbes on the gut barrier function can be readily accessed by implementing the Smurf assays, where a blue or florescent dye is fed to animals to measure the extent of gut leakiness, as the dye leaks from the damaged gut lumen into the body cavity [102,104].

## 3. Exploring the interactions between *Caenorhabditis elegans* genetics and the microbiome

It has become evident in the past two decades that the microbiome plays a critical role in regulating the health of host animals [9]. However, there is potentially enormous complexity in the genetic interactions occurring between a host and its microbiome. We have only begun to understand the capacity of the microbiome to expand the genomic potential of its host, and the genetic and metabolic features of the microbiome that are regulated by the host [105]. In the case of *C. elegans*, both the host and many key microbiome bacteria are genetically amenable, facilitating a systematic and contextual understanding of these genetic interactions.

It has been less than a decade since it was first shown that *C. elegans* assembles its microbes in a deterministic process. We have only begun to understand how *C. elegans* genetics helps shape their microbiome. Recently, it was identified that insulin/insulin growth factor (IGF)-1-like receptor, DAF-2, dictates the gut microbiome composition in *C. elegans* [16]. *Ochrobactrum* spp. are microbiome bacteria known to be abundant and persistent in the *C. elegans* gut, despite being under-represented in nematode-containing environmental samples, implying selective enrichment in the host [26]. Interestingly, natural *C. elegans* variants with high levels of *Ochrobactrum* spp. were found to have high insulin signalling levels, broadly activated host immunity and faster population growth rates. Conversely, variants with low levels of *Ochrobactrum* spp. had lower insulin signalling, higher bacterial diversity, activated stress pathways, and lower population growth rates [16]. This insulin-signalling dependent determination of an *Ochrobactrum*-dominated gut microbiome was validated by knocking down the *daf-2* insulin-like receptor gene*,* that was also dependent on the its downstream transcription factors, DAF-16/FOXO and PQM-1 [16].

These findings identified a novel role of the well-studied insulin signalling pathway in regulating microbiome composition and diversity. However, what remains to be studied is how changes in microbial compositions owing to DAF-2/insulin signalling impacts the longevity and other well-studied life-history traits of *daf-2* mutants, including dauer formation [106]. As reduced insulin/DAF-2 signalling is associated with improved stress resistance and longevity, having a more diverse microbiome might correlate with better health outcomes in *C. elegans* as well. These questions can provide insight into the role that insulin/IGF-1 signalling has in defining the microbial composition of the gut in higher mammals.

We are only beginning to use *C. elegans* genetics to systematically understand different aspects of host–microbiome interactions. *Caenorhabditis elegans* has been a pioneering model organism for over 70 years, studied largely in the context of its interaction with OP50 *E. coli.* It has been used extensively to understand how genetics regulate many organismal phenotypes, including development, neurobiology, stress, ageing and metabolism. With the relatively recent standardization of core microbiomes, there is enormous potential in decoding how the microbiome can impact many well-studied organismal properties. This includes using *C. elegans* to understand the influence of host genes on microbiome or individual microbiota, and its concomitant impact on *C. elegans* life-history traits, longevity paradigms, behaviour and immunity.

In addition, it will be interesting to investigate how different energy and nutrient regulating cellular processes in *C. elegans*, such as autophagy and mitochondrial energetics, influence gut microbiota assembly and composition. Furthermore, unbiased forward genetic screen, or whole-genome mutagenesis screens that select for specific phenotypes, can be easily implemented to identify the underlying host genes and pathways regulating microbiome colonization and adherence [107]. Also, *C. elegans* transcriptomics would capture gene expression changes after colonization by simple or complex microbiota. Together, these studies will probably identify novel microbiome-related functions in known *C. elegans* genes and pathways, as well as novel pathways among the approximately 40% of *C. elegans* genes that currently lack annotation [108].

## 4. Conclusion and future prospects

Since the identification of its natural microbiota, *C. elegans* has emerged as a promising model system to study host–microbiome interactions. Microbiome assembly in *C. elegans* is primarily determined by host genetics, and shaped by other factors such as behaviour, anatomy and abiotic factors, immunity, nutrition and inter-microbial interactions. *C. elegans* microbiota bacteria use diverse metabolic competencies to colonize the gut, probably to ensure diversity in microbial composition. In agreement with this, compared to fast niche occupiers (ruderal strategy), bacteria with competitive or stress tolerating strategies become more prevalent in the *C. elegans* gut [75], which is likely to have an ethological relevance during boom-bust cycles [51]. In addition, these host–microbiome associations are dynamic, and host immunity and tissue homeostasis are critical for maintaining host–commensal interactions [13].

Our current understanding of the *C. elegans* microbiome is mostly limited to bacteria colonizing their gut, owing to the lack of other known microbes such as fungi and viruses colonizing their gut or any other tissues, such as epidermis. In addition, though we can visually resolve bacteria in a tissue-specific manner, resolving them for isolation and characterization is difficult as *C. elegans* are too small for complicated dissections needed to obtain tissue-specific microbiome samples, and in particular the skin microbiome would be more difficult to resolve. However, despite these limitations, *C. elegans* as a microbiome model also has advantages that can pave the way to new biology. For example, one of the major challenges in microbiome research is the inability to culture the vast majority of microbial species outside of their host. *Caenorhabditis elegans* allows for the *in vivo* study of unculturable microbes with as relative ease as any wild-isolated worms found to be colonized with such a microbe can be cultured at a large scale. Microbial preparations can be obtained from colonized worms, similar to the production of microsporidian preparations in *C. elegans* allowing the study of this obligate intracellular pathogen [22].

In addition, the role of *C. elegans* genetics in dictating the microbiota composition is just becoming apparent, forging avenues to systematically study how the genetic regulation of host's behaviour, immunity, diet, gut health and lifespan impacts the microbiome. On the microbial side, bacterial genetics can be used to understand their genomic traits required for host interactions. Also, by examining bacterial gene expression changes after colonizing the *C. elegans* gut, we can identify the microbial genes and metabolites required for colonization and persistence in *C. elegans* gut. Such an unbiased approach is expected to identify genes coding for bacterial mucin-adhesion, pili and fimbriae, and, glycosidases in the case of glyolytic commensals. Moreover, studying the gene expression changes in bacteria with increasing *C. elegans* age can be used to address if there is an active switch from commensal-like traits to pathobiont-like traits in different known microbiome members.

Furthermore, the short generation time of *C. elegans* would allow for the uncovering of the heritable aspects of microbiome bacteria in carefully designed trans-generational studies. Additionally, *C. elegans* gut-microbiome studies can be used to understand inter-tissue crosstalks, as the gut microbiome can be considered as an organ in itself that can interact and influence other organs in the body, including animal behaviours. Currently, one of the major medical goals of the microbiome field is to engineer bacteria with attributes to manage health and disease in humans. Using *C. elegans,* it would be possible to genetically modify commensal bacteria and study their impact on host physiology and disease to understand the probiotic attributes of the modified bacteria. As such, *C. elegans* will continue to be the model of choice to study other heterologous bacteria, including human pathogens and commensals.

## Data Availability

This article has no additional data.
